# Relational, Ethical, and Care Challenges in ALS: A Systematic Review and Qualitative Metasynthesis of Nurses’ Perspectives

**DOI:** 10.3390/brainsci15060600

**Published:** 2025-06-03

**Authors:** Giovanna Artioli, Luca Guardamagna, Nicole Succi, Massimo Guasconi, Orejeta Diamanti, Federica Dellafiore

**Affiliations:** 1 Department of Medicine and Surgery, University of Parma, 43124 Parma, Italy; giovanna.artioli@unipr.it (G.A.); nicole.succi@studenti.unipr.it (N.S.); massimo.guasconi@unipr.it (M.G.); 2 Department of Orthopedics and Traumatology, Istituti Clinici di Pavia e Vigevano S.p.A., 27100 Pavia, Italy; 3 Healthcare Professions, Veneto Institute of Oncology IOV—IRCCS, 35128 Padua, Italy; orejeta.diamanti@iov.veneto.it; 4 Department of Life Science, Health, and Health Professions, Link Campus University, 00165 Rome, Italy

**Keywords:** amyotrophic lateral sclerosis (ALS), nurses’ experiences, palliative care, ethical dilemmas, emotional burden

## Abstract

Background: Amyotrophic lateral sclerosis (ALS) is a progressive neurodegenerative disease that leads to severe functional decline and death, imposing significant physical, emotional, and ethical burdens on patients and healthcare providers. With no curative treatment, ALS care depends on the early and sustained integration of palliative care to address complex and evolving needs. Nurses play a pivotal role in this process, yet their lived experiences remain underexplored. This study aimed to synthesize qualitative evidence on nurses’ experiences in ALS care, with a focus on emotional, ethical, and palliative dimensions. Methods: A meta-synthesis of qualitative studies was conducted using Sandelowski and Barroso’s four-step method. A systematic search across five databases identified eight studies exploring nurses’ experiences with ALS care. Thematic synthesis was applied to extract overarching patterns. Results: Three core themes emerged: (1) *Relational Dimension*: *From challenges to empathy* and *Trust and mistrust*—emphasizing communication barriers and the value of relational trust; (2) *Care Dimension*: *Competence*, *Palliative care needs*, and *Rewarding complexity*—highlighting the emotional demands of care, the need for timely palliative integration, and the professional meaning derived from ALS care; (3) *Ethical Dimension*: *Medical interventionism* and *Patient-centered values*—exploring dilemmas around life-sustaining treatments, patient autonomy, and end-of-life decisions. Conclusion: Nurses in ALS care face complex emotional and ethical challenges that call for strong institutional support and palliative training. Enhancing palliative care integration from diagnosis, alongside targeted education and psychological support, is crucial to improving care quality and sustaining the well-being of both patients and nurses.

## 1. Background

Amyotrophic lateral sclerosis (ALS), also known as Charcot’s disease or Lou Gehrig’s disease, is a progressive neurodegenerative disorder marked by progressive motor neuron degeneration, leading to paralysis and respiratory failure [1]. The ALS incidence rate varies between 0.6 and 3.8 per 100,000 person-years, and its prevalence rates range from 4.1 to 8.4 per 100,000 population. The disease is more common in men, with a male-to-female ratio between 1 and 2, and the mean age of onset is between 51 and 66 years [2]. Globally, ALS affects approximately 5 in 100,000 people annually, with significantly lower incidence in Asia compared to Europe and North America [2]. In Italy, ALS affects an estimated 6000 people, with an incidence rate of 2.5 per 100,000 population annually [3]. The disease typically manifests between the ages of 60 and 79 and is more prevalent among men [4].

Despite limited pharmacological options—such as riluzole and edaravone—ALS remains incurable, requiring multidisciplinary care to manage symptoms and maintain quality of life [5]. Specifically, quality of life (QoL) in ALS is a multidimensional construct and is particularly impacted in several specific areas, including psychological, emotional, and existential well-being. Previous studies have highlighted how factors such as self-efficacy, coping strategies, psychological flexibility, hope, resilience, illness perceptions, guilt, and neuroticism can significantly influence the patient’s experience and perceived QoL [6]. These psychosocial dimensions are often deeply intertwined with the emotional and ethical challenges explored, suggesting the need for targeted attention and support in these specific domains [7].

Additionally, the standard of care relies on a multidisciplinary approach focused on symptom management and quality-of-life preservation [8]. Additionally, comprehensive palliative care from early in the disease trajectory is required in ALS care [5,8]. Palliative care in ALS extends beyond end-of-life support, encompassing anticipatory planning, symptom relief, psychosocial care, and ethical decision-making throughout the disease course. Given the prolonged and complex nature of ALS, integrating palliative principles early and consistently is critical to addressing patients’ and families’ evolving needs [9].

Beyond physical decline, ALS deeply impacts patients’ psychological and emotional health, often causing anxiety, depression, and existential distress [10]. Up to 50% of patients develop frontotemporal dysfunction, further complicating care [6]. The burden also falls heavily on informal caregivers, who face chronic emotional, physical, and financial stress [11]. The high levels of stress, depression, and social isolation experienced by ALS caregivers underscore the need for comprehensive support systems [11].

Nurses play a central role in ALS care, especially in the interdisciplinary palliative care team, delivering not only clinical and palliative support, but also navigating emotionally and ethically complex situations [12]. Their responsibilities span symptom management, patient advocacy, communication, and end-of-life decision-making [12]. Additionally, the progressive and terminal nature of ALS presents significant challenges for nursing professionals. As stated by Gamskjae and colleagues, nurses involved in ALS care experience high levels of emotional distress due to prolonged exposure to patient suffering and the inevitability of death [13]. The emotional burden is further compounded by ethical dilemmas, particularly in cases where patients and families face difficult decisions regarding invasive ventilation, artificial nutrition, and end-of-life care preferences [14].

Although the role of nurses in ALS care is pivotal, current evidence on their lived experiences remains fragmented and lacks comprehensive synthesis. The available literature highlights recurring challenges such as moral distress, compassion fatigue, and burnout, driven by the emotional intensity of caring for patients facing an irreversible decline [14]. Nurses must navigate ethically and emotionally complex situations—balancing honesty and hope, witnessing suffering, and supporting patients and families in highly personal end-of-life decisions. These demands often result in emotional exhaustion and, in some cases, professional withdrawal [15]. Moreover, many nurses report inadequate training and institutional support, particularly in areas such as communication, palliative care planning, and psychosocial support [16].

Given the fragmented nature of current knowledge, a deeper exploration of nurses’ perspectives is essential. Understanding how nurses perceive their role, cope with emotional strain, and respond to the complex realities of ALS care is critical to informing the development of targeted educational strategies, institutional support systems, and evidence-based policies. Centering the nursing perspective is not only vital for improving care quality and ethical decision-making but also for protecting the emotional well-being and professional sustainability of those providing frontline ALS care. In light of these considerations, this study sought to answer the question ‘*What are the experiences and perceptions of nurses providing care to patients with ALS*?’, aiming to synthesize existing empirical research on the perspectives of nurses engaged in the care of individuals with ALS. Through thematic synthesis, this study sought to explain the subjective experiences, emotional responses, and professional challenges encountered by nurses within the context of ALS caregiving.

## 2. Materials and Methods

### 2.1. Study Design

This meta-synthesis was conducted following the four-step method proposed by Sandelowski and Barroso (2007), which involves the systematic retrieval of literature, appraisal of qualitative reports, classification of evidence, and synthesis of findings [17]. This approach ensures a rigorous analytical process that goes beyond simple summary, enabling the generation of interpretive insights. The meta-synthesis represents a secondary level of analysis, reflecting the reviewers’ interpretive reconstruction of the primary researchers’ analyses, which were themselves based on participants reported lived experiences. Consequently, the emergent themes and conceptual categories are substantially removed from the original narratives, constituting a reinterpretation and integration of previous interpretive frameworks [17].

Concurrently, the literature search and review process adhered to the Preferred Reporting Items for Systematic Reviews and Meta-Analyses (PRISMA) guidelines [18], which provide a transparent and structured approach for identifying, screening, and selecting relevant studies. To enhance clarity and transparency specifically for qualitative syntheses, the review also followed the Enhancing Transparency in Reporting the Synthesis of Qualitative Research (ENTREQ, Åstorp, Sweden) statement [19]. We selected ENTREQ over other EQUATOR-endorsed tools (e.g., SRQR, CASP) as it specifically focuses on reporting qualitative evidence syntheses.

Thus, the Sandelowski and Barroso method guided the qualitative synthesis and interpretation, while PRISMA and ENTREQ frameworks ensured systematic and transparent reporting of the literature search and study selection processes. This combination strengthens both the methodological rigor and the analytic depth of the review.

This systematic review was prospectively registered with the International Prospective Register of Systematic Reviews (PROSPERO), under registration number CRD420251028802.

### 2.2. Formulation of the Research Question

The research question was developed using the SPIDER framework to clearly define the key elements of the review and to guide and standardize the search strategy. This question was consistent with the aim introduced in the introduction: ‘*What are the experiences and perceptions of nurses providing care to patients with ALS*?’. The SPIDER elements applied were as follows. Sample: Registered nurses from various clinical contexts and age groups; Phenomenon of Interest: Professional and emotional perceptions during the care of patients with ALS; Design: Qualitative methodologies, including interviews, focus groups, field notes, and approaches based on phenomenology and grounded theory; Design: Qualitative methodologies, including interviews, focus groups, field notes, and approaches based on phenomenology and grounded theory; Evaluation: Lived experiences and subjective perspectives of nurses; Research Type: Qualitative or mixed-method studies [20].

### 2.3. Search Strategy and Eligibility Criteria

We used a combination of free-text keywords and controlled vocabulary (e.g., MeSH in PubMed, Emtree in EMBASE, CINAHL headings), adapted to each database’s indexing system. For instance, in PubMed, we used the MeSH term “Motor Neuron Disease” combined with the free-text term “nurse experience”. Boolean operators (AND, OR) were employed to refine the search, and search strings were adapted to the specific requirements of each database to ensure efficient literature retrieval. Two independent reviewers (LG and MG) systematically searched five databases with comprehensive coverage in the nursing and medical fields: MEDLINE (via PubMed), CINAHL, PsycINFO, EMBASE, and Scopus. Both MeSH terms and free-text keywords were used to build the search strategies, which were explicitly tailored to the syntax of each database.

The inclusion criteria required that studies employed qualitative methodologies, such as interviews, focus groups, grounded theory, or phenomenological approaches. Studies were considered eligible if they explored nurses’ experiences in caring for patients with ALS, and mixed-method studies were included only if the qualitative findings were clearly distinguishable. Furthermore, only peer-reviewed articles written in English, Italian, or Spanish and published up to November 2024 were included in the review. Exclusion criteria were applied to studies using exclusively quantitative methods, as well as to mixed-method designs in which the qualitative component could not be isolated. Studies were also excluded if they did not involve nurses as participants. Additionally, grey literature, conference abstracts, dissertations, books, and non–peer-reviewed materials were not considered.

### 2.4. Data Selection and Extraction

To facilitate the management and organization of the screening process, all citations retrieved from the selected databases were uploaded into Rayyan^®^, a web-based tool designed to support systematic review workflows [21]. Both automated and manual deduplication procedures were applied within the platform to ensure the accurate removal of redundant entries. Subsequently, two independent reviewers (NS and OD) screened the titles and abstracts of all retrieved articles. Potentially relevant studies then underwent a full-text review, conducted independently and blinded to each other’s decisions. Disagreements regarding study eligibility were resolved through discussion; when consensus could not be reached, a third reviewer (GA) was consulted. This deliberative process aimed to enhance the consistency and reliability of the study selection. All articles that passed the abstract screening phase were subjected to multiple, thorough readings to deepen the understanding of each study’s contribution to the synthesis.

### 2.5. Quality Evaluation of the Studies

The methodological quality of the included studies was assessed using the Joanna Briggs Institute (JBI) Critical Appraisal Checklist for Qualitative Research [22]. This tool evaluates the coherence between study objectives, theoretical framework, data collection and analysis methods, and ethical considerations. Each study was assessed against a 10-item checklist, with responses categorized as “Yes”, “No”, “Unclear”, or “Not applicable”. Only studies achieving a score of ≥7 were considered acceptable for inclusion. Quality appraisal was independently conducted by two reviewers (NS and LG), with any discrepancies resolved through consultation with a third reviewer (FD). Studies failing to meet the required quality standards were excluded.

### 2.6. Data Analysis and Synthesis

Data were analyzed following Sandelowski and Barroso’s (2007) approach to qualitative metasynthesis, which aims to develop a higher-level interpretive understanding rather than simply aggregating findings [17]. The synthesis was conducted independently by two reviewers (NS and OD), who performed the coding, categorization, and theme development, following the interpretive method described. Initially, all extracted results from the included studies were read carefully to ensure deep familiarity with the content. Two reviewers independently conducted open coding, identifying meaningful units that reflected participants’ experiences. Codes were developed inductively, staying close to the language used in the original studies. Next, codes were compared across studies to identify recurring ideas and patterns. Similar codes were grouped into categories that captured shared meanings. These categories were iteratively reviewed and refined through discussion and reflection to ensure conceptual clarity and coherence [17]. In the final step, categories were synthesized into overarching themes representing the core dimensions of nurses’ experiences in ALS care. This interpretive process aimed to generate new insights and a cohesive understanding of the phenomenon. Throughout the analysis, reflexivity was maintained to account for the researchers’ influence, and an audit trail documented coding decisions and theme development. Discrepancies were resolved through dialogue, with input from a third reviewer when needed. Themes were supported with representative quotes from the original studies to preserve the participants’ voices and ensure transparency in the interpretive process [17].

## 3. Results

**Figure 1:**
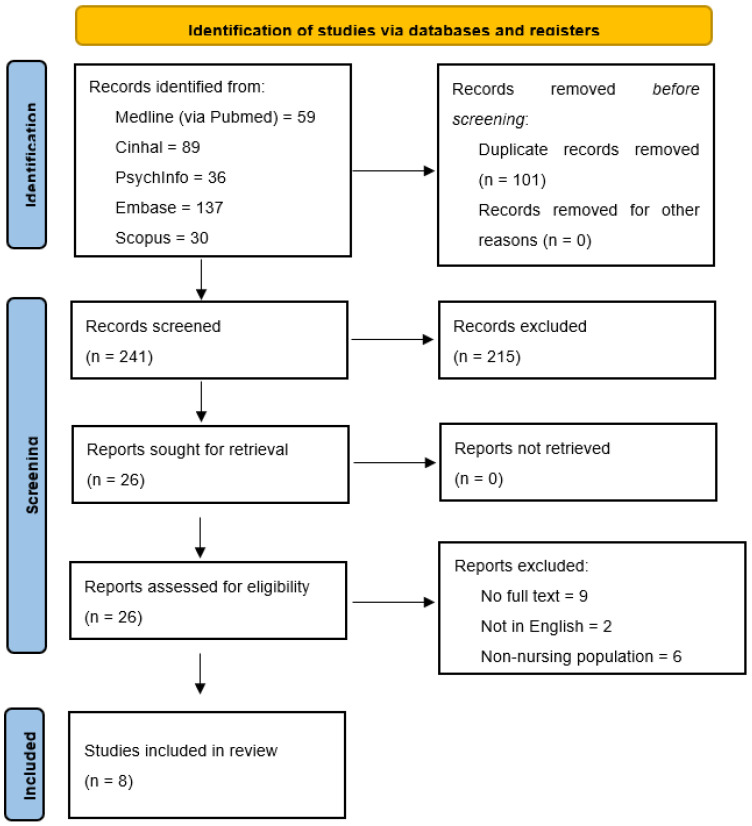
PRISMA flowchart. Source: Page MJ, et al. BMJ 2021;372: n71. doi:10.1136/bmj.n71 [18]. This work is licensed under CC BY 4.0. To view a copy of this license, visit https://creativecommons.org/licenses/by/4.0/ (accessed on 6 May 2025).

The PRISMA 2020 flowchart (Figure 1) reports the selection process of peer-reviewed studies identified through electronic databases. No grey literature was included. Additionally, a completed PRISMA checklist is provided in Supplementary Materials to ensure transparency and facilitate the assessment of methodological rigor. A total of 351 records were initially identified through database searches. After removing duplicates and applying predefined inclusion and exclusion criteria, eight qualitative studies that met the eligibility criteria were selected for full-text review and included in the meta-synthesis. They were qualitative in design, explored nurses’ lived experiences in caring for patients with ALS, and achieved a methodological quality score of ≥7 on the JBI Critical Appraisal Checklist. None of the studies were excluded based on quality, as all met the minimum requirements according to the JBI appraisal tool. A summary of the quality appraisal results is presented in Table 1.

Although we followed the PRISMA flowchart to transparently report the selection process, it should be noted that gray literature was not included in our search strategy. Therefore, the flow diagram reflects only peer-reviewed published studies identified through the selected databases. The rationale for reporting the results according to this specific model derives from the structure suggested by Sandelowski and Barroso (2007) [17], which emphasizes transparency in synthesis and the organization of findings into thematic constructs. For each included study, the following information was systematically extracted and is summarized in Table 2: author(s) and year of publication, country, setting, study aim, design, sample size, results, and main conclusions.

Participants’ ages ranged from 26 to 83 years, and the studies reflect a variety of healthcare systems and cultural backgrounds, including those of Australia, Canada, Japan, Italy, Norway, Sweden, and the United Kingdom. Most studies adopted qualitative methodologies, predominantly semi-structured interviews, with a few employing focus groups. These methods allowed for an in-depth exploration of nurses’ lived experiences, capturing the complex emotional, ethical, and professional dimensions of caring for patients with ALS. The most used analytical approaches were phenomenological and reflexive thematic analysis, both offering nuanced insights into the evolving nurse–patient relationship and the psychosocial challenges of ALS care.

**Figure 2:**
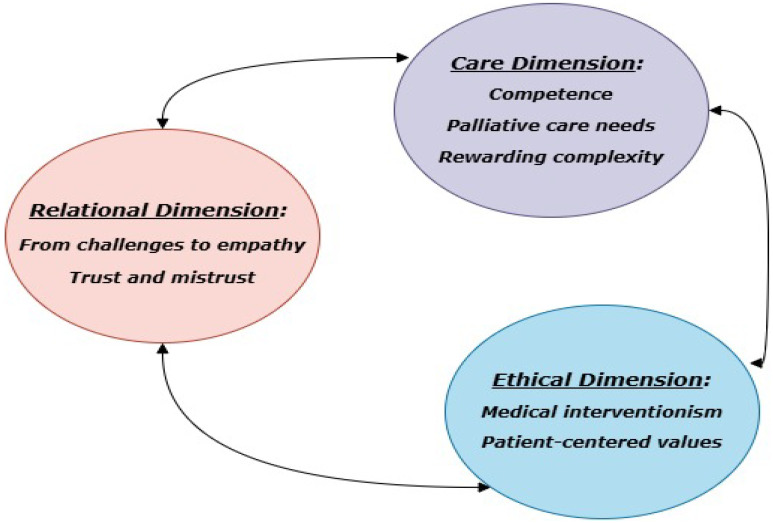
Results.

The results of the synthesis were reported in two complementary formats: a tabular synthesis (Table 3), which includes themes, subthemes, and illustrative quotes from each of the eight included studies; and a narrative synthesis, structured in Section 3.1, Section 3.2 and Section 3.3. The thematic structure presented was derived through an inductive coding process, following the guidelines for qualitative meta-synthesis proposed by Sandelowski and Barroso (2007) [17], and subsequently grouped according to conceptual affinities. Figure 2 visually represents this conceptual framework and guided the organization of the thematic findings.

From the synthesis of the eight studies, three overarching themes emerged: (1) Relational Dimension, with subthemes “From challenges to empathy” and “Trust and mistrust”; (2) Care Dimension, with subthemes “Competence”, “Palliative care needs”, and “The rewarding complexity of care”; and (3) Ethical Dimension, with subthemes “Medical interventionism” and “Patient-centered values” (Figure 2). The themes, subthemes, and illustrative quotes from the eight articles are summarized in Table 3.

All eight studies included in the meta-synthesis met the pre-established inclusion criteria: they were primary, peer-reviewed, qualitative research articles focused on the experiences of nurses caring for patients with ALS. No studies were excluded after the full-text screening phase.

### 3.1. Theme 1. Relational Dimension

Nurses caring for individuals with ALS must navigate complex and evolving relational dynamics, often in contexts marked by communication decline, emotional strain, and significant family involvement. This relational dimension captures how nurses respond to these interpersonal challenges—moving from difficulty to adaptation—while fostering therapeutic relationships essential to holistic care. Two subthemes emerged in this context: From challenges to empathy and Trust and mistrust.

From challenges to empathy. Communication with ALS patients was consistently reported as a significant challenge, especially as the disease progresses, speech becomes impaired or entirely lost. Nurses described how the inability to engage in conventional dialogue made it more difficult to provide emotional support and assess needs, particularly in terminal phases [23]. Nevertheless, many nurses developed alternative strategies that transcended verbal communication, relying instead on empathy, presence, and intuitive understanding. As one nurse explained:

“I think that you don’t need to talk to experience the transpersonal caring relationship… I can say that I am really comfortable, completely comfortable, with saying nothing and simply existing in the presence of someone else […]” [23]. Another nurse described the ongoing challenge of working around communication barriers: “Well, there’s also the psychological side with the person, but there are barriers all the time, because of communication problems […]” [23]. This transition from verbal to empathic and non-verbal modes of interaction was seen as crucial for maintaining human connection and upholding the dignity of care: “You really need to learn to have some empathy and also be quite comfortable talking about end-of-life issues and just how we go about educating some of those things I think is a real challenge because I think some of that has to come through life experience” [24].

Trust and mistrust. Relational dynamics with families and caregivers also emerged as central to the care experience. Nurses highlighted how the establishment of mutual trust could greatly enhance both the quality of care and the emotional well-being of all parties involved [23,25]. When trust was present, caregivers were more likely to welcome nurses into the intimate sphere of the patient’s life and health journey. One nurse reflected: “*Day by day*, *we [were] learning a little bit more about the patient’s routine*, *and day by day*, *she gave us a little more room*, *so she could free herself from the caregiver role and be the spouse again*, *if you will*, *to take some breaks*” [23]. Conversely, in the absence of trust—or when patients and caregivers maintained strict control—nurses often felt scrutinized, stressed, and emotionally burdened: “*She [the individual with ALS] wanted to get it done quickly. Our task was to wash around the PEG*, *which was something that needed to be done*, *get the food and finish the visit. Nothing more. […] And there is very little of that, generally*, *in the services. […] maybe they have realised that we don’t have the time*” [25]. Such tensions, especially when compounded by communication difficulties, could hinder the delivery of truly person-centered care. Building trust also required nurses to validate the emotional experiences of family members, helping them feel acknowledged and supported. As one nurse noted: “*I think the support for them*, *the family*, *can be anything from… that I think above all*, *to show that you see what you see*” [26].

### 3.2. Theme 2. Care Dimension

Caring for individuals with ALS involves increasing technical complexity and significant emotional labor. Nurses often act as key coordinators, balancing clinical responsibilities with personalized support for patients and families. This dimension includes three subthemes—*Competence*, *Palliative care needs*, and *Rewarding complexity*—which highlight how nurses adapt to evolving care demands, navigate institutional limitations, and find meaning in their professional roles.

Competence. Providing care to ALS patients demands advanced technical skills, particularly in managing ventilatory support, gastrostomies, and other complex procedures. Many nurses voiced concerns about insufficient training or lack of preparation, especially when faced with critical respiratory needs or complex symptom management. These gaps often left them feeling underqualified in high-stakes situations: “*Eventually one has to use assistive devices and medical equipment; all personnel need to be able to use that. This requires a high level of skill and you need to feel confident using it. They [persons with ALS] get respiratory problems*, *and you need to suck them for mucus. Using cough assist and BiPAP. To be honest*, *this municipality was not prepared. If there is much uncertainty and insecurity it does not work out. Then it turns into chaos*” [25]. Competence also extended beyond technical know-how, encompassing the ability to support families emotionally and address misinformation: “*Often re-explaining medication management to reassure and correct misunderstandings… especially regarding morphine for respiratory symptoms […]*” [23].

Palliative care needs. The need for early integration of palliative care was a recurring concern. Nurses emphasized that palliative support should begin at diagnosis to help anticipate physical decline and address psychosocial needs. However, in practice, access to specialized palliative services was often delayed until the advanced stages of illness: “*I think it would be really good if the patient could be managed in a palliative care service from the time of diagnosis […]*” [24]. In many cases, families were left to manage care with minimal professional involvement: “*Home care nurses visited him/her once a week or so*, *and the family managed everything else*” [27]. Systemic barriers—such as time constraints and delayed patient acceptance—often made it difficult for nurses to deliver individualized, compassionate care: “*I would say that sometimes it takes weeks for the patient’s acceptance to arrive*, *and also*, *I think*, *for us to accept that ‘they’re there’. Because I really think we want to fight with them.*” [23].

Rewarding complexity. Although physically and emotionally demanding, ALS care was often experienced as deeply meaningful. Nurses acknowledged that certain patients required a disproportionate amount of time and attention, occasionally limiting their ability to care for others: “*You know*, *I have no choice but to be with this patient*, *because he’s the one who takes up most of my time […]*” [23]. Even seemingly simple tasks, like repositioning, could become time-intensive and impact broader workload management: “*Some patients frequently press the nurse call button just for positional changes… this affects our ability to care for other patients*” [28]. Despite these challenges, many nurses found profound professional satisfaction in supporting patients through such a pivotal stage of life. The complexity of ALS care, though taxing, was seen as an opportunity to provide meaningful, high-impact care: “*Well*, *it’s like*, *I saw this as fair. Not equal*, *but it’s what the person needed*” [28].

### 3.3. Theme 3. Ethical Dimension

Ethical challenges are deeply woven into the care of individuals with ALS, particularly in relation to treatment decisions, end-of-life planning, and the preservation of patient autonomy. Nurses are often caught at the crossroads of patient preferences, family expectations, and institutional norms. This ethical dimension is explored through two subthemes—*Medical interventionism* and *Patient-centered values*—which underscore the moral tensions and responsibilities that shape ALS care.

Medical interventionism. Ethical dilemmas were most pronounced in situations where clinical decisions conflicted with the expressed wishes of the patient. Nurses frequently experienced moral distress when treatments were pursued despite a patient’s prior refusal, often due to pressure from family members or conflicting medical opinions: “*The purpose of the wife was to take care of her husband in any condition*, *using any device to stay alive*” [29].

Some described instances where junior physicians initiated aggressive interventions despite ethical concerns and opposition from both the patient and family: “*I find myself even now*, *with patients coming from the emergency room and maybe the family members saying ‘No*, *we don’t want more things interventions’*, *and instead the young doctor can’t get away from it… so we call for resuscitators*, *we administer important drugs*, *we improperly use instruments*” [29]. Nurses involved in the withdrawal of assisted ventilation reported a sense of moral alignment with the decision, while also acknowledging the emotional difficulty of the process: “*Morally and ethically*, *I think it was absolutely the right thing… and I’m really chuffed we somehow pulled it out of the bag and did it so quickly*” [30].

Patient-centered values. The extent to which end-of-life care reflected patients’ values often depended on the presence of clear advance care planning. In its absence, families and healthcare professionals were left to make emotionally fraught decisions with limited guidance: “*We thought that the cognitive decline and the progression of ALS would make it difficult for family caregivers to confirm the patient’s preferences for ventilator use…*” [28]. Professional support from physicians was inconsistent. In some cases, general practitioners refused to participate in ethically sensitive actions, such as ventilator withdrawal: “*She [the GP] said she was going to have absolutely nothing to do with removing the ventilator or touching the settings…*” [30]. Beyond institutional challenges, nurses also expressed concern about how such decisions might be perceived by the public, particularly through the lens of media representation. There was a fear that end-of-life care could be misunderstood or sensationalized: “*If that story had been in the news… if it’s a tabloid*, *they’re not going to want to engage in the ethics of it*” [30].

## 4. Discussion

This meta-synthesis aimed to explore the experiences and perceptions of nurses caring for patients with ALS. The study synthesized diverse qualitative research, highlighting three critical themes: relational, care, and ethical dimensions of ALS nursing. These findings reveal the profound emotional and ethical challenges faced by nurses, emphasizing the importance of empathy, trust, and the complexities of palliative care. This work fills a significant gap in the literature, offering new insights into the often-overlooked emotional burden on healthcare providers in ALS care. The strength of this synthesis lies in its comprehensive approach, which offers a deeper understanding of the nurse’s role in this context. The findings are essential for shaping targeted educational strategies and support systems to improve care and protect nurses’ well-being.

### 4.1. Relational Dimension

The relational dimension highlights the deep emotional connections that often develop between nurses and patients with ALS. Nurses play a crucial role in building and maintaining trust, providing emotional support, and fostering a therapeutic relationship characterized by empathy and presence. These relationships are intensified by the progressive and terminal nature of the disease, which often leads to long-term care scenarios. Nurses reported that their presence is a key source of comfort for patients and families, particularly during the most vulnerable phases of the disease trajectory. However, these close bonds may also increase the emotional burden on nurses, who often experience profound grief and emotional fatigue. Despite these challenges, many nurses find great professional and personal fulfillment in these relationships, as they enable meaningful human connections and reinforce the caring ethos of the nursing profession.

### 4.2. Care Dimension

The care-related dimension encompasses the complex and specialized clinical demands of ALS management. Nurses are required to possess advanced competencies in respiratory and nutritional care, particularly in relation to interventions such as non-invasive ventilation and gastrostomy [31]. The integration of palliative care principles into routine clinical practice is essential throughout the disease trajectory—not only at the end of life. Indeed, early and continuous palliative care discussions are now advocated in major guidelines to support quality of life and facilitate advance care planning [32,33]. Nurses are frequently the first to recognize subtle changes in patients’ health status and are thus critical in identifying appropriate moments to initiate palliative care interventions [34].

Effective multidisciplinary collaboration is another cornerstone of high-quality ALS care. Multidisciplinary teams, including neurologists, palliative care specialists, and allied health professionals, can significantly improve symptom control, patient satisfaction, and even survival [26,35]. Embedding palliative care specialists into these teams strengthens their capacity to manage complex symptoms and navigate difficult conversations [36]. Moreover, evidence indicates that early palliative care integration reduces symptom burden, improves quality of life, and does not increase mortality [37]. Systematic reviews also suggest that some models of home-based palliative care can reduce hospital admissions and overall healthcare costs [38]. These findings reinforce the need for continuing education and support for nurses, whose skills are essential for implementing and sustaining these models of care.

### 4.3. Ethical Dimension

The ethical dimension centers on the moral challenges nurses face while supporting patients through progressive functional decline, decisions about life-prolonging interventions, and end-of-life care. Nurses often find themselves navigating ethically complex situations, including decisions around the initiation or withdrawal of ventilation, gastrostomy, or sedation. These scenarios can generate feelings of moral distress, particularly when patients’ wishes are unclear or when family members’ views conflict with the patient’s autonomy. Furthermore, nurses reported encountering significant gaps in addressing the holistic needs of patients, especially those related to intimacy and spirituality. Patients and their caregivers often express a desire for physical and emotional closeness, which is sometimes overlooked in clinical practice [39].

In line with recent findings, spiritual and existential needs—including nonreligious concerns such as the search for inner peace, meaning, and generativity—are increasingly recognized as crucial yet often unmet components of ALS care [40]. These needs are not only stable over time but are also deeply embedded in the patients’ and caregivers’ lived experiences, regardless of age, setting, or perceived isolation. Nurses are often the first to witness these existential struggles but may feel underprepared to respond appropriately, especially when spiritual needs are expressed in nonreligious or implicit ways. The failure to identify and address these concerns may exacerbate ethical tensions and moral uncertainty, particularly when patients express fear, despair, or loss of meaning [40].

Therefore, the ethical competence of nurses must extend beyond clinical decision-making to include sensitivity to existential suffering and the ability to collaborate with interdisciplinary teams in delivering spiritually responsive care. This includes using validated tools for spiritual needs assessment, documenting such needs systematically, and initiating timely interventions in partnership with chaplains, psychologists, and palliative care specialists. Ethical competence, emotional resilience, and institutional support are essential in helping nurses manage the burden of moral decision-making and avoid burnout. Nurses must be equipped to recognize and respond to these needs, in coordination with interdisciplinary teams, to provide truly person-centered care. Ethical competence, emotional resilience, and institutional support are essential in helping nurses manage the burden of moral decision-making and avoid burnout.

### 4.4. Strengths, Limitations, and Future Research Directions

One of the major strengths of this meta-synthesis lies in its comprehensive approach to synthesizing qualitative studies, offering a deeper and more nuanced understanding of nurses’ experiences in ALS care. By focusing on three core dimensions—relational, care, and ethical challenges—this study provides a holistic view of the emotional and professional burdens faced by healthcare providers. A key strength of this meta-synthesis lies in the rigorous and transparent methodology adopted, notably the adherence to the PRISMA guidelines for systematic reviews and the use of the SPIDER tool for qualitative evidence synthesis. PRISMA ensured structured reporting and replicability, enhancing transparency and completeness in the review process. The SPIDER tool, specifically designed for qualitative studies, allowed a targeted and sensitive search strategy that aligned well with our research question focused on experiences and perceptions. Together, these frameworks enhanced methodological rigor, minimized selection bias, and increased the clarity of data synthesis procedures. However, this work is not without limitations. The synthesis is based on studies from diverse cultural contexts, which may influence the transferability of the results. Moreover, the reliance on qualitative studies, although valuable for depth and detail, limits the generalization of the findings to broader populations.

Future research should aim to develop and test targeted interventions designed to strengthen nurses’ coping strategies when facing emotional, ethical, and relational challenges in ALS care. Longitudinal studies could offer valuable insights into how nurses’ experiences and needs evolve over the trajectory of the disease. Furthermore, interdisciplinary research could explore collaborative care models, integrating the perspectives of physicians, psychologists, social workers, and family caregivers, to better address the complex needs of ALS patients and their families. Comparative studies across different healthcare systems and cultural settings would also be beneficial to identify context-specific best practices and global challenges. Finally, it would be essential to investigate the role of organizational and policy-level factors—such as staffing, training, and emotional support programs—in mitigating burnout and moral distress among nurses involved in ALS care.

Future research could benefit from exploring the impact of hospital organizational factors on nursing care quality through quantitative or mixed-method studies. Additionally, the development and validation of standardized questionnaires to measure nurses’ emotional, physical, and ethical burdens could provide valuable tools to complement qualitative findings and improve understanding of factors affecting patient care quality.

## 5. Conclusions

This study offers crucial insights into the often-invisible struggles of nurses caring for ALS patients, exposing a landscape marked by emotional exhaustion, ethical dilemmas, and professional vulnerability. The synthesis not only sheds light on the urgent gaps in support and training but also calls for a paradigm shift in how healthcare systems recognize and sustain their frontline caregivers. Investing in nurses’ wellbeing is not optional—it is essential for the dignity of both patients and professionals. Future research must drive innovative strategies to empower nurses, protect their resilience, and enhance the quality of ALS care globally. This meta-synthesis successfully addressed the research question by synthesizing the qualitative evidence on nurses’ lived experiences and perceptions in caring for ALS patients. The findings provide a comprehensive understanding of the multifaceted challenges they encounter and offer valuable guidance for clinical practice and future research.

## Figures and Tables

**Table 1:** JBI quality evaluation.

	Authors	ITEM 1	ITEM 2	ITEM 3	ITEM 4	ITEM 5	ITEM 6	ITEM 7	ITEM 8	ITEM 9	ITEM 10	Inclusion
Beyermann et al., 2023	NS	Y	Y	Y	Y	Y	U	U	Y	U	Y	Included
	LG	Y	Y	Y	Y	Y	U	U	Y	U	Y	
Daneau et al., 2023	NS	Y	Y	Y	Y	Y	N	U	Y	Y	Y	Included
	LG	Y	Y	Y	Y	Y	N	U	Y	Y	Y	
Phelps et al., 2022	NS	Y	Y	Y	Y	Y	N	N	Y	Y	Y	Included
	LG	Y	Y	Y	Y	Y	N	N	Y	Y	Y	
Ushikubo et al., 2021	NS	Y	Y	Y	Y	Y	N	U	Y	Y	Y	Included
	LG	Y	Y	Y	Y	Y	N	U	Y	Y	Y	
Cipolletta and Reggiani 2021	NS	Y	Y	Y	Y	Y	U	N	Y	Y	Y	Included
	LG	Y	Y	Y	Y	Y	U	N	Y	Y	Y	
Ushikubo, 2018	NS	Y	Y	Y	Y	Y	U	U	Y	Y	Y	Included
	LG	Y	Y	Y	Y	Y	U	U	Y	Y	Y	
Lerum et al., 2017	NS	Y	Y	Y	Y	Y	Y	Y	Y	Y	Y	Included
	LG	Y	Y	Y	Y	Y	Y	Y	Y	Y	Y	
McConigley et al., 2013	NS	Y	Y	Y	Y	Y	U	U	Y	Y	Y	Included
	LG	Y	Y	Y	Y	Y	U	U	Y	Y	Y	

Legend: Y = Yes; N = No; U = Unclear.

**Table 2:** Data extraction.

Study	Country	Setting	Study Aim	Study Design	Sample Size (Sex)	Themes	Conclusions
Beyermann et al., 2023	Sweden	Specialized palliative home care	To explore RNs’ experiences of supporting families of ALS patients	Qualitative study	11 nurses (10 F; 1 M)	(i) “To support in an increasingly difficult everyday life”; (ii) “To support in emotionally challenging situations”	RNs play a vital role in emotional and daily life support for families.
Daneau et al., 2023	Canada	Home care, hospital, palliative care homes	To explore nurses’ experience in end-of-life ALS care	Qualitative multiple-case study	24 nurses (20 F; 4 M)	(i) identifying the end-of life period, (ii) communication issues, (iii) supporting the need for control, (iv) accompanying in the fight culture, and (v) the extent of the need for care	ALS care requires better staffing, recognition of family involvement, and resources.
Phelps et al., 2022	UK	Hospitals and hospices	To explore experiences of relatives and healthcare professionals during withdrawal of assisted ventilation in ALS.	Retrospective qualitative study	26 healthcare professionals (Not specified)	(i) Emotional intensity, (ii) ethical/legal ambiguity, (iii) lack of professional guidance, (iv) teamwork importance	Withdrawal of ventilation requires clearer guidelines and training to reduce distress and ensure safe, ethical practice.
Ushikubo et al., 2021	Japan	Home care, hospital, public health centers	To identify practical strategies for nurses caring for ALS patients	Qualitative study	58 nurses (57 F; 1 M)	(i) “Patients’ strong persistency on specific requirements for nursing assistance in their daily lives”, (ii) “Patients’ problematic behaviors toward nurses”, and (iii) “Struggles in communicating with and understanding patients’ wishes.”	Practical tools and shared experiences improve confidence and care quality.
Cipolletta & Reggiani, 2021	Italy	Various healthcare services and patient associations	To explore end-of-life care after the legal introduction of advance directives.	Qualitative study	24 healthcare professionals (16 F; 8 M)	(i) Lack of organization, (ii) collaboration and continuity on the part of healthcare services and professionals, (iii) a lack of information on palliative care, (iv) advance care planning, and (v) advance directives	Legal introduction not sufficient without structural and training support.
Ushikubo et al., 2018	Japan	Home care with non-invasive ventilation	To explore circumstances and signs of approaching death in ALS	Retrospective qualitative study	6 nurses (Not specified)	(i) Difficulties with knowing about approaching death, (ii) several signs and symptoms of knowing about approaching death, (iii) importance of feeling prepared and provision of palliative care to die at home, (iv) death caused by accident, and (v) fate determined by the caregiver’s ability	Recognizing death signs is crucial for timely and preferred end-of-life care.
Lerum et al., 2017	Norway	Home care	To explore challenges in managing ALS in the community	Qualitative study	18 healthcare professionals (Not specified)	(i) building relationships with those giving and receiving care in the home; (ii) preventing caregiver burnout and breakdown; (iii) providing tailored care; (iv) ensuring good working conditions in patients’ homes; (v) recruiting and retaining qualified nursing assistants.	Home-based ALS care is emotionally challenging and requires better support.
McConigley et al., 2013	Australia	Community and hospital settings	To examine nurses’ perceptions of caring for ALS patients	Qualitative study	31 healthcare professionals (Not specified)	(i) Just One Step Ahead; (ii) Expertise in MSD and bespoke communication	Palliative care should start early; team communication is critical.

**Table 3:** Themes, subthemes, and quotes.

Theme	Subtheme	Quote
Relational dimension	From challenges to empathy	“I think that you don’t need to talk to experience the transpersonal caring relationship. You can experience sadness and pain without necessarily having to express it verbally. When we know that it’s the spirit that’s not ok, we deduce the rest and we feel the sorrow together. At the beginning of my training, it was important for me to do something, to say something, but with the passage of time, I realized that silence has its place in a therapeutic relationship. Now, I can say that I am really comfortable, completely comfortable, with saying nothing and simply existing in the presence of someone else and getting them to express their suffering in ways other than talking” (Daneau et al., 2023)
		“Well, there’s also the psychological side with the person, but there are barriers all the time, because of communication problems. You know? What is a good therapeutic approach to take with someone who doesn’t communicate 100% verbally? That’s what gets complicated too, because you can’t understand them” (Daneau et al., 2023)
		“You really need to learn to have some empathy and also be quite comfortable talking about end-of-life issues and just how we go about educating some of those things I think is a real challenge because I think some of that has to come through life experience” (McConigley et al., 2013)
	Trust and mistrust	“Day by day, we [were] learning a little bit more about the patient’s routine, and day by day, she gave us a little more room, so she could free herself from the caregiver role and be the spouse again, if you will, to take some breaks” (Daneau et al., 2023)
		“She [the individual with ALS] wanted to get it done quickly. Our task was to wash around the PEG, which was something that needed to be done, get the food and finish the visit. Nothing more. Never any talk, never sitting down and sharing a cup of coffee or something like that. Not by either of them [the two with ALS in that region]. And there is very little of that, generally, in the services. […] maybe they have realised that we don’t have the time”(Lerum et al., 2017)
		“I think the support for them the family can be any thing from that I think above all, to show that you see what you see” (Beyermann et al., 2023)
Care dimension	Competence	“Eventually one has to use assistive devices and medical equipment; all personnel need to be able to use that. This requires a high level of skill and you need to feel confident using it. They [persons with ALS] get respiratory problems, and you need to suck them for mucus. Using cough assist and BiPAP. To be honest, this municipality was not prepared. If there is much uncertainty and insecurity it does not work out. Then it turns into chaos” (Lerum et al., 2017)
		“Often re-explaining medication management to them because morphine affects breathing too. Sometimes people forget that, so it’s about repeating it, reteaching it, and reassuring them. You know, some people are afraid to take morphine for breathing, but it helps so much. So, it’s about reassuring them, removing the fears and false beliefs if you will” (Daneau et al., 2023)
	Palliative care needs	“I think that, yes, it’s a terrible disease, but I think that we have the means—or the capacity—as nurses to do wonderful things with these patients as well: you just have to take the time. Of course, experience helps, but you have to take the time” (Daneau et al., 2023)
		“Home care nurses visited him/her once a week and experienced difficulties assessing symptom aggravation” (Ushikubo, 2018)
		“I would say that sometimes it takes weeks for the patient’s acceptance to arrive, and also, I think, for us to accept that ‘they’re there’. Because I really think we want to fight with them. I think there’s this pattern in our heads, and I would also like to say that, in terms of diet, for example, the step from ‘normal’ to ‘bite-sized’ is not so bad, but when you go from ‘bite-sized’ to ‘minced’, then the step seems to be HUGE, and no one wants to take it. It’s like putting them [the patient] in front of a fait accompli” (Daneau et al., 2023)
	Rewarding complexity	“You know, I have no choice but to be with this patient, because he’s the one who takes up most of my time. But my stroke patient, who is also being tube fed and is panicking because he doesn’t understand the instructions even though his brain is working, I neglect him. So, I always neglect two or three of them because of the ALS workload” (Daneau et al., 2023)
		“Some patients frequently press the nurse call button to request fine, millimetric repositioning no matter what time” (Ushikubo et al., 2021)
		“Well, it’s like, I saw this as fair. Not equal, you understand—it’s not the same level of attention, but it’s what the person needs” (Ushikubo et al., 2021)
Ethical dimension	Medical Interventionism	“The purpose of the wife was to take care of her husband in any condition, using any device to stay alive” (Cipolletta and Reggiani 2021)
		“I find myself even now, with patients coming from the emergency room and maybe the family members saying ‘No, we don’t want more things interventions’, and instead the young doctor can’t get away from it… so we call for resuscitators, we administer important drugs, we improperly use instruments” (Cipolletta and Reggiani 2021)
		“We have asked for help for this… that is, help us understand how can we relate to these people here. How can you tell someone ‘your life is about to end’?” (Cipolletta and Reggiani 2021)
		“Morally and ethically, I think it was absolutely the right thing and I’m really chuffed we somehow pulled it out of the bag and did it so quickly …” (Phelps et al., 2022)
	Patient-centered values	“We thought that the cognitive decline and the progression of ALS would make it difficult for family caregivers to confirm the patient’s preferences for ventilator use…” (Ushikubo et al., 2021)
		“She [the GP] said she was going to have absolutely nothing to do with removing the ventilator or touching the settings or…which made it very difficult because I couldn’t guarantee that I was going to be available around the clock to do it, whereas she could have been, more so, or she could have, perhaps, interacted with me” (Phelps et al., 2022)
		“If that story had been in the news, how would that have been perceived? And would it be the case that some local newspaper would then suddenly say ‘palliative care nurses can now help you die’. And you know, if it’s a tabloid they’re not going to want to engage in the ethics of it, yeah. So I think that was something that was on my mind a little bit” (Phelps et al., 2022)

## Supplementary Materials

The following supporting information can be downloaded at: https://www.mdpi.com/article/10.3390/brainsci15060600/s1, Table S1: PRISMA 2020 Checklist.
